# Correlation between Sonographic Features and Central Neck Lymph Node Metastasis in Solitary Solid Papillary Thyroid Microcarcinoma with a Taller-Than-Wide Shape

**DOI:** 10.3390/diagnostics13050949

**Published:** 2023-03-02

**Authors:** Shun-Ping Chen, Xin Jiang, Wu-Wu Zheng, Yin-Li Luo

**Affiliations:** Department of Ultrasonography, The First Affiliated Hospital of Wenzhou Medical University, Wenzhou 325000, China

**Keywords:** thyroid, microcarcinoma, ultrasonography, taller than wide, central lymph node metastasis, extrathyroidal extension

## Abstract

**Purpose:** This study aimed to investigate the correlation between sonographic features and central neck lymph node metastasis (CNLM) in solitary solid papillary thyroid microcarcinoma (PTMC) with a taller-than-wide shape. **Methods:** A total of 103 patients with solitary solid PTMC with a taller-than-wide shape on ultrasonography who underwent surgical histopathological examination were retrospectively selected. Based on the presence or absence of CNLM, patients with PTMC were divided into a CNLM (*n* = 45) or nonmetastatic (*n* = 58) group, respectively. Clinical findings and ultrasonographic features, including a suspicious thyroid capsule involvement sign (STCS, which is defined as PTMC abutment or a disrupted thyroid capsule), were compared between the two groups. Additionally, postoperative ultrasonography was performed to assess patients during the follow-up period. **Results:** Significant differences were observed in sex and the presence of STCS between the two groups (*p* < 0.05). The specificity and accuracy of the male sex for predicting CNLM were 86.21% (50/58 patients) and 64.08% (66/103 patients), respectively. The sensitivity, specificity, positive predictive value (PPV), and accuracy of STCS for predicting CNLM were 82.22% (37/45 patients), 70.69% (41/58 patients), 68.52% (37/54 patients), and 75.73% (78/103 patients), respectively. The specificity, PPV, and accuracy of the combination of sex and STCS for predicting CNLM were 96.55% (56/58 patients), 87.50% (14/16 patients), and 67.96% (70/103 patients), respectively. A total of 89 (86.4%) patients were followed up for a median of 4.6 years, with no patient having recurrence as detected on ultrasonography and pathological examination. **Conclusions**: STCS is a useful ultrasonographic feature for predicting CNLM in patients with solitary solid PTMC with a taller-than-wide shape, especially in male patients. Solitary solid PTMC with a taller-than-wide shape may have a good prognosis.

## 1. Introduction

Papillary thyroid carcinoma is the most common pathological subtype of thyroid carcinoma [1]. Its incidence has increased in recent years, likely owing to the improvement of examination techniques such as ultrasonography [2]. Papillary thyroid microcarcinoma (PTMC) refers to papillary thyroid carcinoma of ≤10 mm in size [3] and accounts for >30% of all newly diagnosed thyroid cancer cases [2,4]. PTMC is considered indolent, and patients are often classified as having a low risk owing to a favourable prognosis. However, it is noteworthy that a characteristic of PTMC is the tendency for lymph node metastasis. The most common site of PTMC lymph node metastasis is central neck lymph node metastasis (CNLM), and the incidence of CLNM in PTMC is not negligible [5]. Patients with PTMC with CNLM may require thyroidectomy and central lymph node dissection, whereas those without CNLM may require only observation, follow-up, or minimally invasive treatment such as thermal ablation [6]. Therefore, developing an effective method for accurate prediction of CNLM is crucial for guiding clinical decision-making in PTMC.

At present, ultrasonography is the first-line imaging modality for examining CLNM in patients with PTMC, whether directly or indirectly. The direct method includes preoperative detection of abnormal metastatic neck lymph nodes that often appear with calcification, cystic necrosis, hyperechogenicity, and the absence of an echogenic hilum [7,8,9]. However, the ability of preoperative ultrasonography to identify CLNM is limited owing to the overlying thyroid gland, and these lymph nodes do not usually appear abnormal on preoperative ultrasonography or inspection at the time of surgery. Therefore, direct detection of CLNM on preoperative ultrasonography may have low predictive accuracy [10]. This disadvantage led to the increased use of the indirect method of ultrasonography, which includes the examination of PTMC features and clinical characteristics for predicting CLNM [11,12,13,14,15,16]. Several studies have examined clinical predictive and/or ultrasonographic factors for assessing CLNM in patients with PTMC [11,12,13,14,15,16]. However, most studies were focused on multiple PTMC or multiple coincidence with solitary PTMC [11,12,13,14,15,16]. A few studies have analysed differences in sonographic features of solitary solid PTMC between patients with CLNM and those without metastasis. We hypothesised that the shape of PTMC may correlate with the occurrence of CLNM. Therefore, in this study, we investigated the correlation between sonographic features and CLNM in solitary solid PTMC with a taller-than-wide shape. The taller-than-wide shape was defined as an anteroposterior diameter greater than the transverse diameter, longitudinal diameter, or both, which may also indicate a special shape of solitary solid PTMC in a three-dimensional (3D) view, as described in a previous study [17].

## 2. Methods

### 2.1. Patient Selection

This retrospective study was approved by the Review of Ethics Committee in Clinical Research of the First Affiliated Hospital of Wenzhou Medical University (Approval Date: 27 April 2022; Approval Number: KY2022-R045). The requirement for informed patient consent for this retrospective review was waived.

A total of 4463 patients with solitary solid PTMC underwent surgical and histopathological examination after thyroid ultrasonography at our hospital from September 2009 to October 2012, and their records were reviewed retrospectively. The inclusion criteria were as follows: (a) patients with solitary solid PTMC confirmed through surgical histopathological examination; (b) patients who underwent total thyroidectomy or near-total thyroidectomy or lobectomy with (or without) isthmectomy; (c) patients who underwent central neck lymph node dissection (CNLD) surgery; (d) patients in whom the time interval between ultrasonography and surgery was ≤3 months. The exclusion criteria were as follows: (a) patients who did not undergo CLND; (b) ultrasound data were incomplete, inconclusive, or both.

In a previous study, we analysed the data of patients with thyroid nodules smaller than or equal to 10 mm in size with a taller-than-wide shape to determine the effectiveness of the taller-than-wide shape in predicting PTMC [17]. However, in the present study, we used the data of patients with PTMC with a taller-than-wide shape to determine the effectiveness of clinical and ultrasound findings in predicting CLNM and prognosis. Solitary solid PTMC was defined as PTMC that satisfied any one of the following two conditions: (a) the presence of an isolated and single solid PTMC; (b) the presence of an isolated solid PTMC associated with single or multiple benign thyroid nodules in the contralateral thyroid lobe (or in the isthmus) confirmed on surgical histopathological examination.

Based on the abovementioned definition, 403 patients were initially selected, of which 296 patients were excluded for the following reasons: the size of the tumour was not consistent with that determined through pathological examination (*n* = 15), CLND was not performed (*n* = 158), skip metastases were found (*n* = 6), ultrasound images were unclear (*n* = 2), and a taller-than-wide shape was not observed (*n* = 119). Eventually, 103 patients (24 men and 79 women; age, 26–69 years (average: 45.00 ± 8.82 years)) were included in this study. A total of 85 patients had an isolated and single solid PTMC, and 18 patients had an isolated solid PTMC in only one thyroid lobe. The interval between surgery and preoperative ultrasonography was 6 days in all patients (interquartile range (IQR), 2.25–11 days; range, 1–48 days).

Based on the presence or absence of central neck lymph node metastasis (CNLM), the patients were divided into the CNLM (*n* = 45, with 3 patients having concomitant lateral neck lymph node metastasis) or nonmetastatic (*n* = 58) group, respectively. The interval between surgery and preoperative ultrasonography in the CNLM and nonmetastatic groups was 7 days (IQR, 2–10 days; range, 1–26 days) and 6 days (IQR, 3–11 days; range, 1–48 days), respectively. No significant differences were observed in the interval between the two groups (*p* = 0.777). The patients were followed-up postoperatively for detecting recurrence on neck ultrasonography (mainly neck lymph nodes and the thyroid bed) every 3–6 months. If patients experienced any discomfort in the neck postoperatively, ultrasonography was performed irrespective of whether the interval between surgery and ultrasonography was ≥3–6 months. Data on demographic characteristics, clinical history, and outcomes were obtained from the electronic medical information database of the hospital.

### 2.2. Ultrasound Imaging

Thyroid ultrasonography was performed by experienced sonographers based on a standard procedure. A 7–15-MHz linear array transducer (HDI 5000; Philips Medical Systems, Bothell, WA, USA), an 8–15-MHz linear array transducer (Acuson Sequoia; Siemens Medical Solutions, Mountain View, CA, USA), or a 5–12-MHz linear array transducer (iU22; Philips Medical Systems, Bothell, WA, USA), (Mylab 90 or DU6; ESAOTE; Genoa, Italy) was used for the scan. All US images were obtained before surgery, and sonographer (S.-P.C.) was blinded to the surgical and histopathological results before reviewing the ultrasonographic images and reports; therefore, the sonographer had no knowledge of the final evaluation of thyroid nodules.

The taller-than-wide shape was defined as an anteroposterior diameter greater than the transverse diameter, longitudinal diameter or both [17]. Ultrasonographic features, including position, location, size, echogenicity, shape, margin, calcification, and suspicious thyroid capsule involvement, were compared between the two groups. The positions used to describe the location of PTMCs were the left lobe, right lobe, and isthmus. The location of PTMCs in the thyroid lobe was classified as upper, middle, and lower third. The size of tumours was measured via ultrasonography in three dimensions (longitudinal, anteroposterior and transverse), and the maximal diameter was considered the tumour size. Echogenicity was classified as hyperechogenicity, isoechogenicity, hypoechogenicity, or marked hypoechogenicity. When the echogenicity of PTMC was similar to that of the thyroid parenchyma, it was defined as isoechoic. Marked hypoechogenicity was defined as echogenicity that was less than that of the surrounding strap muscle. Margins were classified as regular (round or ovoid) or irregular (microlobulated (the presence of many small lobules on the surface of a tumour)). Calcifications were classified as microcalcifications or macrocalcifications. Microcalcifications were defined as calcifications that were ≤ 1 mm in size and were visualised as tiny punctate hyperechoic foci, either with or without acoustic shadows. If tiny bright reflectors with a clear-cut comet tail artefact were detected on conventional ultrasonography, they were considered colloids. Macrocalcifications were defined as hyperechoic foci of >1 mm in size. If both types of calcifications (macrocalcifications, including rim calcifications, combined with microcalcifications) were observed, the sample was classified as having microcalcifications. The internal components of PTMC were defined as solid, mixed, or cystic. PTMC with mixed components had both solid and cystic components.

Suspicious thyroid capsule involvement was defined as PTMC abutment or disruption of the thyroid capsule and included two conditions: capsular abutment with or without disruption of the thyroid capsule (either the anterior or posterior thyroid capsule). Capsular abutment was defined as the lack of intervening thyroid tissue between PTMC and the thyroid capsule. Capsule disruption was defined as the loss of the anterior (or posterior) perithyroidal echogenic line at the site of contact, with PTMC as described in previous studies [18,19,20,21,22].

The detection of a suspicious recurrence sign on postoperative neck ultrasonography included assessment of the neck lymph node and thyroidectomy bed. The suspicious recurrence sign in neck lymph nodes was described as neck lymph nodes with calcification, cystic necrosis, hyperechogenicity, and the absence of an echogenic hilum [7,8,9]. The suspicious recurrence sign in the thyroidectomy bed was described as follows: (a) hypoechoic or (and) hyperechoic thyroidectomy bed lesion size larger than 6 mm or (b) lesion size smaller than 6 mm with microcalcification [23,24]. The final locoregional recurrence was diagnosed via ultrasonography plus pathological examination when necessary. Images of all included lesions were retrospectively reviewed independently by one author (S.-P.C., with 28 years of ultrasound experience and special expertise in thyroid ultrasound).

### 2.3. Surgery and Pathological Examination

The patients underwent total thyroidectomy (*n* = 10), near-total thyroidectomy (*n* = 26), lobectomy with isthmectomy (*n* = 27), or lobectomy (*n* = 40). All patients also underwent bilateral CLND (*n* = 3) or unilateral CLND (*n* = 100). None of the patients had undergone radioiodine treatment before or after surgery. Thyroid malignancy was confirmed based on histopathological examination. Specimens were fixed in buffered formalin, embedded in paraffin, and stained with haematoxylin and eosin for histological analysis. All specimens and pathological reports were evaluated by faculty members in the Department of Pathology at our institution. Additionally, Hashimoto’s thyroiditis and nodular goitre were diagnosed based on the final pathological results.

### 2.4. Statistical Analysis

The MedCalc statistical software (version 11.4.1; Frank Schoonjans, Mariakerke, Belgium) was used for statistical analysis. Categorial variables were expressed as numbers and (or) percentages and compared using the chi-square test and Fisher’s exact test. Normally distributed data were expressed as the mean ± standard deviation (SD) and compared using a two-sample *t*-test. Non-normally distributed data were expressed as the median (IQR) and compared using the Mann–Whitney *U* test. Statistical significance was set at *p*-values of <0.05. The predictive performance of significant parameters was examined in terms of sensitivity, specificity, accuracy, positive predictive value (PPV), and negative predictive value (NPV).

## 3. Results

### 3.1. Clinical Characteristic

The size of tumours in the 103 patients with solitary solid PTMC was 4–10 mm (7.59 ± 1.65 mm). The number of lymph nodes in the CNLM group was 2 (IQR, 1–3; range, 1–7). A total of 89 (86.4%) patients were followed-up for 54.8 months (4.6 years) (IQR, 14.0–109.4 months; range, 2.3–133.4 months), and 14 patients (3 in the CNLM group and 11 in the nonmetastatic group) were lost to follow-up. The number of ultrasound examinations performed per patient in those 89 patients ranged from one to 22 (mean, 5.75). Among the 89 patients, 70 and 43 patients were followed-up for more than 1 and 5 years, respectively. The interval between surgery and the last follow-up in the two groups was not significantly different (47.6 months (13.7–108.5 months); range, 2.7–123.6 months versus 74.3 months (14.4–114.4) months; range, 2.3–133.4 months; *p* = 0.138). According to the findings of ultrasonography and pathological examination, recurrence was not observed in any patient (only 1 patient had a suspicious enlarged neck lymph node with the presence of an echogenic hilum; after the patient underwent FNAB, the pathological result was inflammation).

### 3.2. Correlation between Sonographic Features and Central Neck Lymph Node Metastasis in Solitary Solid PTMC with a Taller-than-Wide Shape

**Figure 1:**
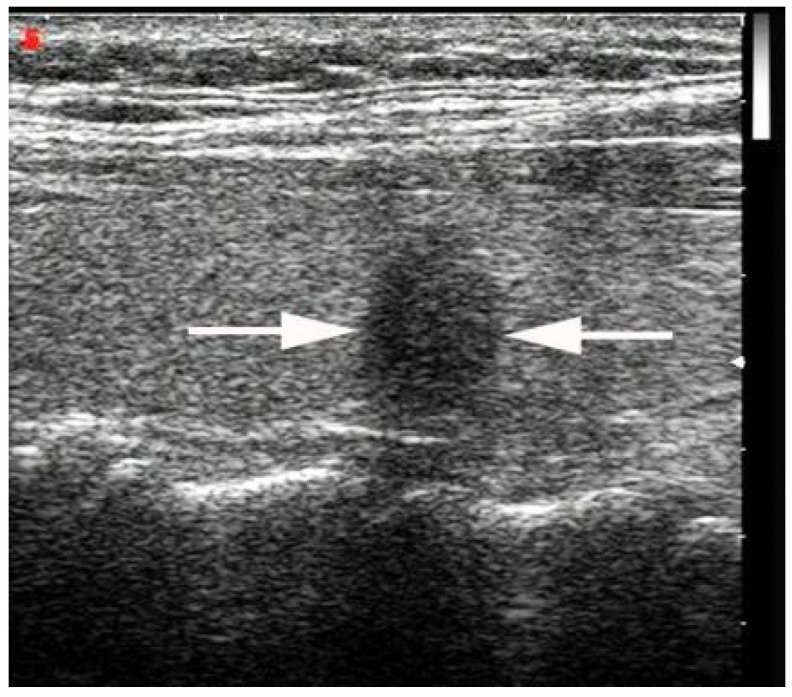
A 48-year-old woman with a PTMC with a taller-than-wide shape in the left thyroid lobe without suspicious thyroid capsule involvement sign (arrows). The patient underwent left central neck lymph node dissection, and the surgical–histopathologic results showed the absence of central neck lymph node metastases. PTMC: papillary thyroid microcarcinoma.

**Figure 2:**
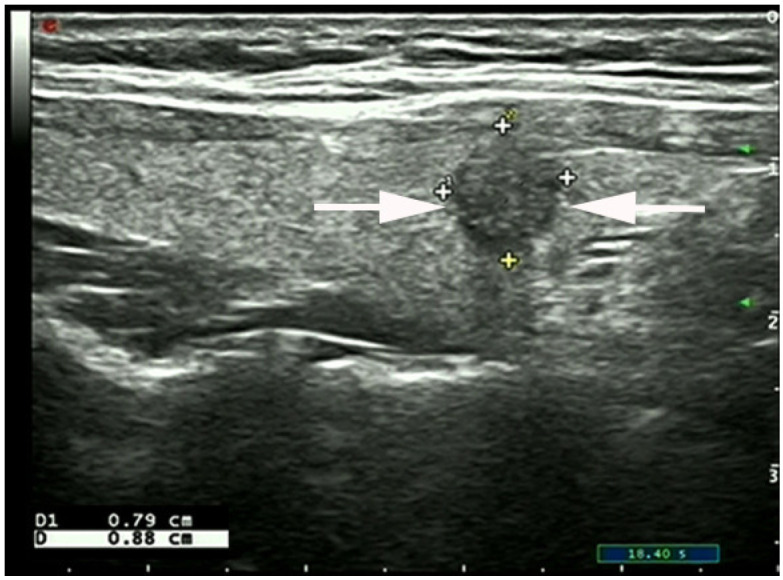
A 39-year-old woman with PTMC with a taller-than-wide shape in the right thyroid lobe with suspicious thyroid capsular involvement sign (PTMC involving the anterior thyroid capsule, arrows; + indicated measurement of the PTMC). The patient underwent right central neck lymph node dissection, and the surgical–histopathologic results showed the presence of two central neck lymph nodes metastases. PTMC: papillary thyroid microcarcinoma.

**Figure 3:**
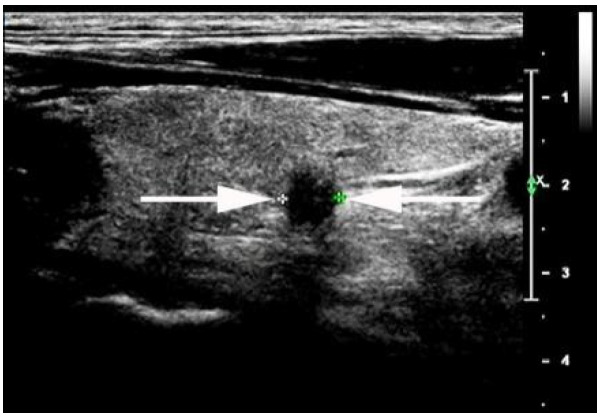
A 36-year-old man with PTMC with a taller-than-wide shape in the right thyroid lobe with suspicious thyroid capsule involvement sign (PTMC involving the posterior thyroid capsule, arrows; + indicated measurement of the PTMC). The patient underwent right central neck lymph node dissection, and the surgical–histopathologic results showed the presence of one central neck lymph node metastases. PTMC: papillary thyroid microcarcinoma.

Significant differences were observed in sex and the presence of STCS between the two groups (*p* < 0.05) (Table 1, Figure 1, Figure 2 and Figure 3). However, no significant difference was observed in age, position, location, size, echogenicity, margin, and calcification between the two groups. Additionally, the incidence of Hashimoto’s thyroiditis and nodular goitre did not significantly differ between the CLNM and nonmetastatic groups.

### 3.3. Effectiveness of Sex (Male) or (and) STCS in Predicting Central Lymph Node Metastasis in Solitary Solid PTMC with a Taller-than-Wide Shape

The specificity and accuracy of the male sex for predicting CNLM were 86.21% (50/58 patients) and 64.08% (66/103 patients), respectively. The sensitivity, specificity, PPV, and accuracy of STCS for predicting CNLM were 82.22% (37/45 patients), 70.69% (41/58 patients), 68.52% (37/54 patients), and 75.73% (78/103 patients), respectively. The specificity, PPV, and accuracy of the combination of sex and STCS were 96.55% (56/58 patients), 87.50% (14/16 patients), and 67.96% (70/103 patients), respectively (Table 2). However, no significant difference was found in diagnostic accuracy among the three predictive methods (*p* > 0.05).

## 4. Discussion

If the size of the thyroid tumour is small, the detection of extrathyroidal extension and CNLM is very important for surgical decision-making and determining the extent of surgical resection. Ultrasonography is a useful tool for detecting the presence or absence of extrathyroidal extension in PTMC. The most common ultrasonographic feature for detecting extrathyroidal extension in PTMC is capsular abutment or disruption of the thyroid capsule [18,19,20,21]. The presence of extrathyroidal extension is associated with CNLM in PTMC [11,25,26,27,28]. Therefore, capsular abutment or disruption of the thyroid capsule may be associated with CNLM. In this study, we defined capsular abutment or disruption of the thyroid capsule as a suspicious thyroid capsule involvement sign (STCS) to examine the relationship between STCS and CNLM in patients with solitary solid PTMC with a taller-than-wide shape.

The presence of STCS was associated with the occurrence of CNLM in solitary solid PTMC with a taller-than-wide shape. STCS had higher sensitivity (82.22% (37/45 patients)), specificity (70.69% (41/58 patients)), and accuracy (75.73% (78/103 patients)) for detecting CNLM. To the best of our knowledge, this finding has not been reported in previous studies. The results of this study are partly consistent with those of previous studies [11,25,26,27,28], indicating that STCS is associated with CNLM. Additionally, CNLM has been associated with the male sex, younger age, larger tumour size, and multifocal tumours in PTMC [11,25,27,28]. However, our result did not agree with previous studies [5,22] that STCS was associated with lateral neck lymph node metastasis in PTMC patients but not associated with CNLM. This discrepancy may be attributed to the presence of tumours with a taller-than-wide shape in all patients with PTMC in this study. However, the true cause for this difference warrants further investigation.

Although the taller-than-wide shape is an ultrasonographic feature for predicting PTMC in smaller thyroid nodules with higher accuracy (approximately 68.3% (643/942) [17], it is not affected by the size of malignant tumours and the tilt and orientation of the probe [29]. The actual taller-than-wide shape may reflect a special shape of PTMC in the three-dimensional (3D) view. As tumours grow in a 3D orientation, solitary solid tumours with taller-than-wide shape were considered a special 3D-shape of PTMC to investigate its biological behaviour (tumour growth and invasion) in the present study. In our previous study [17], ultrasonography revealed that tumours grew in a taller-than-wide shape in many patients with PTMC (approximately 49.4% (254/514)). This study revealed that tumours were invasive in some patients (35.92% (37/103)) and subsequently led to CNLM in a taller-than-wide manner (anterior or posterior capsular abutment or disruption). This finding indicates that although tumours in some patients with PTMC mainly grow in a taller-than-wide manner (anteroposterior orientation), their invasion or CNLM may also occur in a taller-than-wide orientation. Therefore, thyroid microcarcinoma initially grows in a taller-than-wide manner; as it continues to grow and its size increases, it may reach the anterior or posterior thyroid capsule, leading to invasion or CNLM in a taller-than-wide (anteroposterior) orientation. However, owing to a relatively small cohort, the results of this study warrant further verification.

The male sex was identified as another predictor of CNLM in solitary solid PTMC with a taller-than-wide shape in this study, which is consistent with the results of a previous study [5,11,27,28,30]. The combination of the male sex and STCS had higher specificity (96.55%) and PPV (87.50%) for predicting CNLM. This result indicates that male patients with PTMC with a taller-than-wide shape with STCS are at high risk for CNLM, which has not been reported in previous studies.

In this study, patients were followed-up postoperatively for approximately 11 years (median, 4.6 years). No patient had signs of recurrence on ultrasonography plus pathological examination, which may indicate a good prognosis irrespective of the presence or absence of CNLM. The present results are consistent with the previous study, in that for postoperative follow-up the PTMC patients were all doing well and were free of any clinical local recurrence or distant metastases [31]. Meanwhile, ultrasonography is a reliable method for monitoring the recurrence of thyroid carcinoma [24]. In this study, it was performed by experienced sonographers in our hospital; therefore, the results of postoperative ultrasonography performed during the follow-up are reliable. Additionally, no patient with CNLM had PTMC recurrence after the postoperative follow-up in this study, which is not consistent with previous studies demonstrating that the presence of lymph node metastasis in patients with PTMC is associated with an increased risk of regional recurrence [32,33,34]. This difference may only be attributed to the inclusion of patients with solitary solid PTMC with a taller-than-wide shape in this study. However, the present results are consistent with previous studies, showing that surgery in PTMC has excellent outcomes [35].

This study has important clinical implications. First, we considered the taller-than-wide shape as a special shape of PTMC in the 3D view to observe its biological behaviour. Although a previous study [36] reported that 3D thyroid ultrasonography can be used to diagnosis thyroid carcinoma, 3D ultrasonography is used to assess only irregular margins for diagnosing carcinoma and not to assess the growth behaviour of PTMC for predicting CNLM. Second, if lower-risk thyroid microcarcinoma with a taller-than-wide shape is detected during follow-up, the presence of STCS may be used as an indicator for predicting CNLM to guide clinical decision-making.

This study has some limitations. First, capsular abutment with or without disruption was not subdivided as in previous studies [18,19,20,21,22] because of the small cohort. We will include the subclassification in our future study. Second, other suspicious capsular involvement signs, such as tracheal invasion and recurrent laryngeal nerve [20] invasion, were not assessed in this study and warrant further investigation. Third, colour Doppler or contrast-enhanced ultrasonography was not used to determine capsular abutment with or without disruption. Lastly, thyroid hormone and genetic testing, such as assessment of thyroid-stimulating hormone (TSH) level and the BRAF gene which may be helpful for prediction CNLM [35,37], were not performed for predicting CNLM. We will consider these aspects in our future study.

## 5. Conclusions

STCS is useful for predicting CNLM in patients with solitary solid PTMC with a taller-than-wide shape, especially in male patients. Solitary solid PTMC with a taller-than-wide shape, irrespective of the presence or absence of CNLM, may indicate a good prognosis. The results can be used for investigating thyroid microcarcinoma biological behaviour in a 3D view and can guide individual treatment strategies.

## Figures and Tables

**Table 1:** Correlation between sonographic features and central neck lymph node metastasis in solitary solid PTMC with a taller-than-wide shape (*n* = 103).

Parameters	CNLM Group (*n* = 45)	Nonmetastatic Group (*n* = 58)	*p* Value
Age (years)	26–69 (43.35 ± 9.95)	31–64 (46.26 ± 7.68)	0.096
Gender			0.021
Male/female (*n*)	16/29	8/50	
Position			0.968
Left/right/isthmic (*n*)	17/28/0	22/36/0	
Location			
Upper pole (*n*)	9	11	0.876
Middle pole (*n*)	22	31	0.796
Lower pole (*n*)	14	16	0.707
Size (mm)	4–10 (7.82 ± 1.68)	4–10 (7.40 ± 1.61)	0.195
Echogenicity			
Hyper/Isoechogenicity (*n*)	0/0	0/0	
Hypo echogenicity (*n*)	35	39	0.484
Mark hypo-echogenicity (*n*)	10	19	0.340
Margin			1
Regular (*n*)	0	0	
Irregular (*n*)	44	59	
Calcification (*n*)	23	27	0.742
Microcalcification (*n*)	20	24	0.770
Macrocalcification (*n*)	3	3	0.739
Without calcification (*n*)	22	31	0.796
STCS (*n*)	37	17	0.0002
Anterior capsule involved (*n*)	15	7	0.018
Posterior capsule involved (*n*)	22	10	0.004
Hashimoto’s thyroiditis (*n*)	7	8	0.757
Nodular goiter (*n*)	4	4	0.765

PTMC: papillary thyroid microcarcinoma; CNLM:central neck lymph node metastasis; STCS: suspicious thyroid capsule involvement sign.

**Table 2:** Effectiveness of sex (male) or (and) STCS in predicting central lymph node metastasis in solitary solid PTMC with a taller-than-wide shape (*n* = 103).

Parameters	Sensitivity (%)	Specificity (%)	PPV (%)	NPV (%)	Accuracy (%)
Male (*n*)	35.56 (16/45)	86.21 (50/58)	66.67 (16/24)	63.29 (50/79)	64.08 (66/103)
STCS (*n*)	82.22 (37/45)	70.69 (41/58)	68.52 (37/54)	83.67 (41/49)	75.73 (78/103)
Male + STCS (*n*)	31.11 (14/45)	96.55(56/58)	87.50 (14/16)	64.37 (56/87)	67.96 (70/103)

STCS: suspicious thyroid capsule involvement sign; PTMC: papillary thyroid microcarcinoma; PPV: positive predictive value; NPV: negative predictive value.

## Data Availability

Research data are available on request from the corresponding author.
